# Hand-Rearing, Release and Survival of African Penguin Chicks Abandoned Before Independence by Moulting Parents

**DOI:** 10.1371/journal.pone.0110794

**Published:** 2014-10-22

**Authors:** Richard B. Sherley, Lauren J. Waller, Venessa Strauss, Deon Geldenhuys, Les G. Underhill, Nola J. Parsons

**Affiliations:** 1 Animal Demography Unit and Marine Research Institute, University of Cape Town, Rondebosch, Western Cape, South Africa; 2 Bristol Zoological Society, Bristol Zoo Gardens, Bristol, United Kingdom; 3 CapeNature, Hermanus, Western Cape, South Africa; 4 Southern African Foundation for the Conservation of Coastal Birds, Bloubergrant, Western Cape, South Africa; University of Sussex, United Kingdom

## Abstract

The African penguin *Spheniscus demersus* has an ‘Endangered’ conservation status and a decreasing population. Following abandonment, 841 African penguin chicks in 2006 and 481 in 2007 were admitted to SANCCOB (Southern African Foundation for the Conservation of Coastal Birds) for hand-rearing from colonies in the Western Cape, South Africa, after large numbers of breeding adults commenced moult with chicks still in the nest. Of those admitted, 91% and 73% respectively were released into the wild. There were veterinary concerns about avian malaria, airsacculitis and pneumonia, feather-loss and pododermatitis (bumblefoot). Post-release juvenile (0.32, s.e.  = 0.08) and adult (0.76, s.e.  = 0.10) survival rates were similar to African penguin chicks reared after oil spills and to recent survival rates recorded for naturally-reared birds. By December 2012, 12 birds had bred, six at their colony of origin, and the apparent recruitment rate was 0.11 (s.e.  = 0.03). Hand-rearing of abandoned penguin chicks is recommended as a conservation tool to limit mortality and to bolster the population at specific colonies. The feasibility of conservation translocations for the creation of new colonies for this species using hand-reared chicks warrants investigation. Any such programme would be predicated on adequate disease surveillance programmes established to minimise the risk of disease introduction to wild birds.

## Introduction

The conservation status of the world's seabirds is poor with c. 47% of species showing population declines and c. 28% occupying positions in the IUCN Red List's threatened categories [1]. In many cases, species face numerous threats, not all of which are well understood in form or function. This highlights the need for further research to improve seabird conservation [2], but also the importance of management actions that can reduce mortality and sustain populations in the short-term [1].

**Figure 1:**
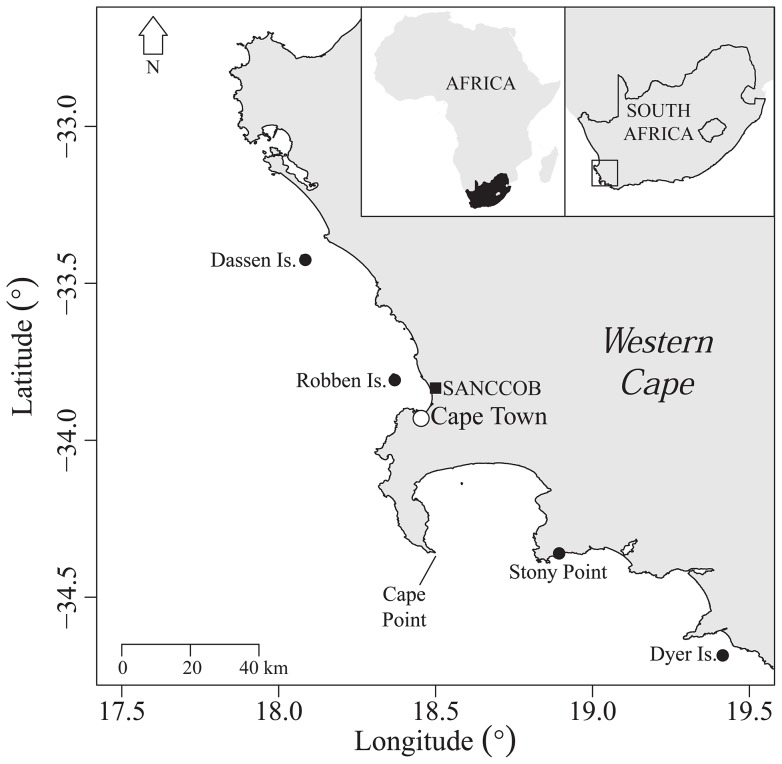
Map of the Western Cape, South Africa, showing the locations of the main African penguin breeding colonies (black circles) mention in the text and the location of SANCCOB (black square) in relation to Cape Town (white circle).

The African penguin *Spheniscus demersus* is ‘Endangered’ following a decrease in the global population of >70% between 2001 and 2013 [3], [4]. Decreases in the Western Cape of South Africa (Figure 1) conform to an altered distribution of their main prey species, sardine *Sardinops sagax* and anchovy *Engraulis encrasicolus* [3], [5]. Adult survival, juvenile survival and breeding productivity of African penguins have been influenced by the availability these two forage fish species [3], [6]–[9] and competition with the local purse-seine fishery has been noted [3], [10]. In addition, growth rates and body condition of chicks at Robben Island decreased between 2004 and 2009 [11]–[13], while fledging periods increased concurrently in apparent response to a decline in the availability of sardine [8]. Spatial management of the fishery has been recommended [3], [8]–[10] and the potential benefits of alternative approaches are being investigated [10], [14].

Concurrently, conservation efforts are focused on strategies to increase breeding success, such as providing artificial nests [15], and to reduce mortality at breeding colonies, for example by rehabilitating oiled and injured adults [16] and their chicks abandoned as a result [16], [17]. Chicks hand-reared after catastrophic oil spills had survival and recruitment rates analogous to naturally-reared cohorts [17], [18] and reproduced successfully once they entered the breeding population [17]. On that basis, a number of African penguin chicks are hand-reared each year at the Southern African Foundation for the Conservation of Coastal Birds (SANCCOB), Cape Town. These chicks may be removed from the wild during the breeding season because they have been orphaned or abandoned by their parents following flooding of their nest site, building operations or the parents being removed for rehabilitation after being oiled [16]. In addition, at the end of the breeding season, some adults may enter moult with chicks still present in the nest [16]. African penguins usually make short foraging trips (<24 hours, [10]) and leave their chicks unattended when feeding conditions are poor (the post-guard phase) [19]. However, moulting penguins are without adequate waterproofing and must fast for c.21 days [20]; unfledged chicks would thus starve in the nest [21]. Here, we use the term ‘abandoned’ to indicate situations where chicks are no longer being provisioned prior to independence, rather than temporary abandonment that occurs naturally in penguins during the post-guard phase [22].

From 2001 to 2005, small numbers (24–99) of abandoned African penguin chicks were retrieved annually from Robben and Dyer Islands and sent to SANCCOB for hand-rearing (Table S1). However, in 2006 and 2007, large numbers (>400) of chicks were abandoned at Dyer Island between September and December, as their parents entered moult. This paper is a case study of the interventions made in 2006 and 2007 to hand-rear these chicks and considers the conservation merit of rearing penguin chicks abandoned prematurely by moulting parents.

## Methods

In the Western Cape, penguins breed from February to September [23] and predominately moult between September and January, once chicks have fledged [24]. The penguin colonies at Dyer Island, Robben Island and Stony Point (Figure 1) were checked regularly for signs of abandoned chicks from the end of the breeding season. Abandoned chicks, identified by appearance and behaviour (apparently low mass relative to structural growth, “hollow” abdomens, lethargy, peck wounds on head and neck), were removed from all three sites and sent to SANCCOB to be hand-reared.

### Chick removals from Dyer Island

At Dyer Island, most adults moult from October to December [24] and do so in in groups within the breeding colony (LJW pers. obs.). The colony was monitored for signs of abandoned chicks from September each year. In 2006, a large proportion of the breeding adults at Dyer Island commenced moult while chicks were still present in nests (Table S2). The managing authority was concerned about the impact that regular approaches into the colony to search for abandoned chicks would have on adult moulters, with birds showing signs of stress at a distance of 20–30 m. It was thus decided to remove chicks *en masse* in both 2006 and 2007 based on four considerations: (1) one operation would minimise disturbance to moulting adults; (2) the timing of moult is highly synchronised at Dyer Island [12], so the remaining chicks would likely be abandoned when parents ultimately commenced moult; (3) hand-reared chicks could potentially boost the breeding population in three to five years' time, depending on juvenile survival and recruitment processes [25], [26]; (4) the poorer the condition of a chick when it reached the rehabilitation centre, the smaller the chances for successful rearing and release.

At Dyer Island, penguins form small, localised sub-colonies. Sub-colonies were slowly surrounded by 4–5 people to prevent adult birds, especially moulters, from moving off, while one person captured the chicks by hand. The chicks were sorted by size into indoor holding pens and gavaged 60 ml electrolyte solution after capture and again before removal to the mainland if kept overnight. The chicks were transported in aerated boxes by boat to the mainland (c. 0.5 hour) and then to SANCCOB by truck (c. 3 hours). In 2006, chicks were removed in large groups and were generally transported to SANCCOB the day after being removed from their nests. In 2007, daily capture numbers were smaller and chicks were transported to SANCCOB on the capture date.

### Chick removals from Robben Island and Stony Point

At Robben Island, the colony was monitored from the end of October and at Stony Point the colony was monitored in November and December. Abandoned chicks were captured from nests by hand on an individual basis or in small groups. There were placed in aerated boxes and transported to SANCCOB the same day by truck (c. 2 hours) from Stony Point and by ferry (c. 0.5 hour) and truck (c. 0.5 hour) from Robben Island.

### Hand-rearing procedures

On arrival at SANCCOB, chicks were grouped into stages of development based on their weight and the level of down present ([11], [27], Appendix S1) and their condition was estimated by “habitus”, scored from 1–4 (weak to strong; Appendix S1) [16].

Chicks were reared following guidelines based on Turner and Plutchak [28]. Chicks were given formula (liquidised fish and vitamin mixture), fluids and whole fish. Veterinary treatment requirements, changes in mass and waterproofing of feathers were evaluated on a weekly basis [16]. Blood samples (haematocrit, total serum protein and blood smears) to evaluate blood parasites, anaemia and systemic inflammatory response were taken weekly or fortnightly. Both flies and mosquitoes were abundant during the chick-rearing period; the netting surrounding the centre at the time was inadequate to exclude insects. Insecticides were used in the pens and applied locally to the birds' heads to help prevent flies and mosquitoes. Various fly traps and fly control products were also employed.

On live birds, conditions such as airsacculitis and pneumonia, avian pox, bumblefoot and feather-loss disorder were diagnosed based on clinical symptoms and lesions only. On birds that died, avian malaria was diagnosed on macroscopic pathology lesions together with positive blood and/or kidney impression smears [29]. Most other diagnoses were determined from macroscopic pathology lesions only. Fungal airsacculitis and pneumonia was differentiated from bacterial cases on the presence of fungal plaques and mats and was not specifically identified to species level. When birds died, the carcase was refrigerated immediately and post-mortem examination conducted on c. 85% of cases within four days. Histopathology and other tests were not routinely performed, except in cases where the cause of death could not otherwise be determined.

### Release and resighting data

Juvenile penguins that met the criteria outlined by Parsons and Underhill [16] were released ashore at Dyer or Robben Islands or else at sea near to Robben Island. Movement of juvenile penguins is extensive [30] and breeding at non-natal colonies occurs [6], [26]. It was thus not deemed vital to return chicks to their natal site. Of those released, 511 were marked with flipper bands from the 2006 cohort and 190 from the 2007 cohort (Table S3).

As part of routine monitoring carried out at African penguin colonies, searches were made for banded individuals and band numbers from throughout the species range (Namibia and South Africa) were reported to a central database (see [6]). The records from this database covering the period 1 January 2007 to 31 December 2012 were searched for resightings.

### Ethics statement

Capture, transportation, rearing, diagnostic screening, care and release of the birds were carried out by SANCCOB on behalf of the Western Cape Nature Conservation Board (CapeNature) and the then Department of Environmental Affairs and Tourism (DEAT, now the Department of Environmental Affairs) under permits (Reference No. V1/1/5/1) issued by DEAT according to the Sea Birds and Seals Protection Act No. 46 of 1973 and the Marine Living Resources Act No. 18 of 1998. SANCCOB is a registered veterinary practice with the South African Veterinary Council (registration number FCO02/5650) and blood samples were taken by a state registered veterinarian to ensure that the birds were fit to be released and were not carrying any diseases that might be introduced to the wild population. Stainless steel flipper bands were applied under license from the South African Bird Ringing Unit (SAFRING) and according to the guidelines approved by the Banding Forum and the Animal Ethics Committee of the DEAT [31].

### Statistical analyses

We estimated survival (*φ*), encounter (or resighting) (*ρ*), and recruitment (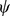) probabilities using multistate mark-recapture models (e.g. [32]). We considered three states; ‘alive as a non-breeding individual’, ‘alive and confirmed breeding’, and ‘dead’ and three events; ‘not encountered’, ‘encountered as a non-breeding individual’ and ‘encountered as a breeder’, which were conditional on the states (see Appendix S2). We implemented our multistate models in a hidden Markov models framework [33] using program E-SURGE v1.9.0 [34] and tested for goodness-of-fit using U-CARE v2.2.3, which indicated little evidence for overdispersion (*ĉ* = 1.11). Parameter estimates are given ±1 standard error (s.e.), with 95% confidence intervals (95% CI) computed from the Hessian matrix.

We developed a set of candidate models that assumed survival probabilities to depend on age, encounter probabilities to be either constant or to vary with time, and recruitment probabilities to depend on age (years after release), time, or be constant across time. Due to sparse resighting data, we did not attempt to estimate time-dependent survival, or to estimate separate survival parameters for the two release cohorts. For the age effects on survival, we distinguished between juveniles (first year after release) and adults (all subsequent years; [6]). For recruitment probabilities, we modelled three age categories, 0−1 years old, 1−2 years old, and >2 years old as African penguins usually breed for the first time at 3 years of age or older [25]. Model selection was performed using the Akaike's Information Criterion adjusted for small sample size and overdispersion (QAICc, [35]).

## Results

In total, 841 and 481 chicks were removed from the three colonies in 2006 and 2007 respectively (Table 1). At Dyer Island, 19 chicks were collected between 18 September and 15 October 2006, prior to the decision to remove chicks *en masse*. Between 16 and 21 October 2006, 668 chicks were captured at Dyer Island on three separate days and transported to SANCCOB. In 2007, the decision to remove all abandoned chicks from Dyer Island was taken on 27 October and 427 chicks were collected. An additional 201 chicks were admitted to SANCCOB from the other two colonies across the two years (Table 1).

**Table 1:** Numbers of African penguin chicks admitted to and released from SANCCOB by colony in 2006 and 2007.

Year	Colony	Admissions	Releases	Release rate	Mean ± SD duration
2006	Robben Island	113	90	80%	35±21
	Dyer Island	694	647	93%	45±16
	Stony Point	34	29	85%	42±18
2007	Robben Island	7	3	43%	25±8
	Dyer Island	427	324	76%	48±22
	Stony Point	47	24	51%	47±25
**Total**		**1322**	**1117**	**84%**	**45±19**

The mean ± standard deviation (SD) duration (in days) of stay in rehabilitation for the released birds is also shown.

### Hand-rearing success

The abandoned chicks were generally underweight for their age [15] and many were not yet losing their down, indicating that they were at least 20 days from fledging [18]. In 2006 and 2007 respectively, 6% and 20% of chicks from Dyer Island were small to medium downy chicks, for Stony Point the corresponding values were 6% and 2%, while none of the birds from Robben Island were small to medium downy chicks.

The chicks were reared in 2006 for a mean of 44 days (range: 11–127 days) for those that were released and 36 days (range: 0–88 days) for those that died. In 2007 rearing lasted a mean of 48 days (range: 15–130 days) for those released and 50 days (0–158 days) for those that died (Table 2 and 3). In both years, chicks that died had a lower habitus on admission than those that were released (2006: χ^2^ = 76.0, p<0.001; 2007: χ^2^ = 19.2, p<0.001; Table 2).

**Table 2:** The habitus of African penguin chicks admitted to SANCCOB in 2006 and 2007.

Habitus	2006			2007		
	Admissions	Releases	Mean ± SD duration	Admissions	Releases	Mean ± SD duration
1	29	16	58±16	25	11	59±11
2	140	113	53±21	173	116	57±25
3–4	672	637	42±15	283	224	43±20
**Total**	**841**	**766**	**44±17**	**481**	**351**	**48±22**

Habitus is scored from 1–4, with one being weak and four being strong (Appendix S1, [16]). The mean ± standard deviation (SD) duration (in days) of stay in rehabilitation is also shown for those birds that were released.

**Table 3:** Causes of death of abandoned African penguin chicks admitted to SANCCOB in 2006 and 2007.

Cause of death	2006			2007		
	N	Deaths	Mean ± SD duration	N	Deaths	Mean ± SD duration
Abscess on heart	1	1.3%	(28)	–	–	–
Airsacculitis and pneumonia	16	21.3%	41±32	23	17.6%	41±31
Fungal airsacculitis and pneumonia	5	6.6%	31±15	3	2.3%	34±24
Multiple organ infection	8	10.5%	33±29	1	0.8%	(52)
Pododermatitis (Bumblefoot)	1	1.3%	(84)	1	0.8%	(46)
Enteritis	–	–	–	3	2.3%	59±28
Blind	1	1.3%	(47)	–	–	–
Nervous symptoms	2	2.6%	48±52	–	–	–
Avian malaria	27	35.5%	48±26	77	59.2%	58±28
Weak, emaciated chick	11	14.7%	7±5	11	8.5%	10±9
Tubed down trachea	2	2.6%	10±6	1	0.8%	(96)
Died during transport	–	–	–	7	5.4%	47±3
Undetermined	1	1.3%	(8)	3	2.3%	54±34
**Total**	**75**		**36±29**	**130**		**50±30**

The mean ± standard deviation (SD) duration (in days) in rehabilitation for individuals in each cause of death category is also shown. Where only one individual died in any category, the duration of stay (days) for that individual in given in parentheses.

In 2006 and 2007, 114 chicks (14%) and 112 chicks (23%) respectively were found to be positive for avian malaria *Plasmodium* spp. (Table S4). Positive birds were treated according to a set of basic treatment protocols (Appendix S1). Those that were released took 20% longer in 2006 and 95% longer in 2007 than all chicks to reach the conditions for release. Malaria was diagnosed as the cause of death for 36% of deaths in 2006 and 59% in 2007 (Table 3).

The second main cause of death was bacterial airsacculitis and pneumonia (Table 3), which can spread from the lungs to infect other organs. No specific aetiological diagnosis was made. Fungal airsacculitis and pneumonia caused 7% of deaths in 2006 and 2% in 2007 (Table 3). Birds diagnosed as “chesty” (laboured breathing, crackly lung noises on auscultation and coughing) were treated with a course of systemic antibiotics (Appendix S1) and nebulised in an enclosed box with a disinfectant. Attempts were made to isolate “chesty” birds, although there was a lack of space when there were large numbers of birds in the facility. Antifungal treatment was also given if there was no response to the antibacterial treatment (Appendix S1).

One bird was euthanized due to blindness caused by avian pox (Table 3). Lesions occurred around the eyes, the ceres, the beak, inside the mouth and occasionally on the feet of the chicks that contracted the disease [36]. The pox lesions were debrided and treated locally with antibiotic eye cream. When swelling occurred around the eyes, the penguins were also treated with systemic antibiotics and anti-inflammatories (Appendix S1). The lesions usually healed after three weeks; in severe cases, scarring caused a smaller eye opening.

In both years, a number of chicks also contracted pododermatitis (bumblefoot; Table 3). Lesions were treated with topical antibiotics and severe cases were also treated with systemic antibiotics and anti-inflammatories (Appendix S1). In 2007, bandages were applied as cushioning to provide some relief to the birds when standing. One bird was euthanased each year due to bumblefoot (Table 3). A feather-loss disorder also occurred in both years, delaying hand-rearing significantly, but did not cause any mortality. These results are discussed in detail by Kane et al. [37].

### Release, survival and recruitment rates

In 2006, 766 hand-reared penguins were released (91% of admissions) and in 2007, 351 chicks were released (73% of admissions, Table S3). Of those released with flipper bands, 92 (13%) were resighted by 31 December 2012. Twelve individuals were confirmed as breeding, all from the 2006 cohort, and 22 others were resighted at breeding age. Of the breeding birds, six were at Dyer Island, three were at Robben Island, two at Stony Point and one at Dassen Island (Table S5). They all originated from Dyer Island (Table S5).

**Figure 2:**
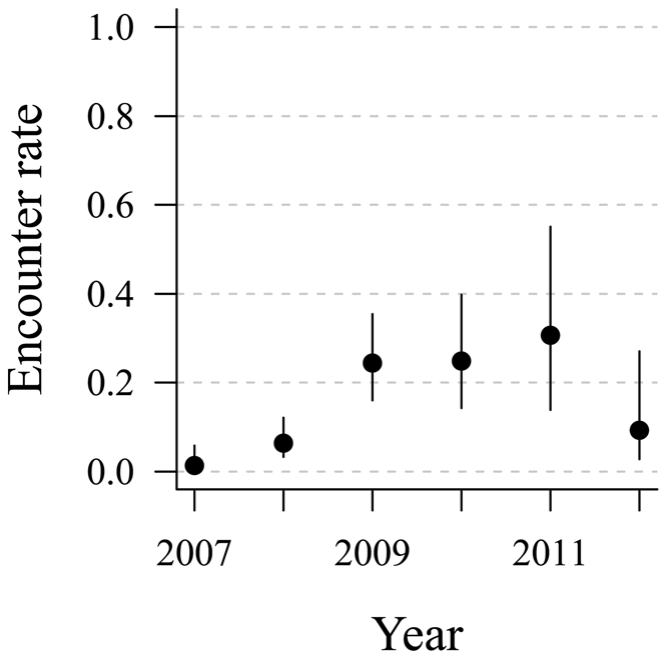
Time-dependent encounter (or resighting) probabilities for banded, hand-reared African penguins released by SANCCOB in 2006 and 2007. Resightings were made over the period 2007 to 2012. Encounter probabilities are based on model 2, Table 4. Error bars show the 95% confidence intervals.

Model selection on the resighting data favoured the model with a constant recruitment probability and time-dependent encounter rates (Model 2, Table 4). Apparent survival was 0.32±0.08 (95% CI: 0.18–0.49) in the first year after release (juvenile survival) and 0.76±0.10 (0.51–0.90) in subsequent years (adult survival). Encounter rates were low initially at 0.01±0.01 (0.00–0.06) in 2007 and 0.06±0.02 (0.03–0.12) in 2008, but increased to 0.31±0.11 (0.14–0.55) in 2011, before falling back in 2012 (Figure 2). The recruitment probability was 0.11±0.03 (0.06–0.19) and there was no support for a change in this parameter over time or within the age structure we identified (Table 4).

**Table 4:** Model selection results for mark-recapture modelling of hand-reared African penguins released by SANCCOB in 2006 and 2007.

Model No.	Model structure	*K*	Deviance	QAICc	ΔQAICc	*w*
2	*φ*(*a*)*ρ*(*t*)*ψ*(*c*)	11	1000.05	1022.37	0	0.82
1	*φ*(*a*)*ρ*(*t*)*ψ*(*a*)	14	996.87	1025.38	3.01	0.18
3	*φ*(*a*)*ρ*(*t*)*ψ*(*t*)	20	993.12	1034.15	11.78	0.00
5	*φ*(*a*)*ρ*(*c*)*ψ*(*c*)	6	1054.41	1066.51	44.15	0.00
4	*φ*(*a*)*ρ*(*c*)*ψ*(*a*)	9	1051.24	1069.45	47.09	0.00
6	*φ*(*a*)*ρ*(*c*)*ψ*(*t*)	15	1047.49	1078.07	55.70	0.00

The model components were survival (*φ*), encounter (*ρ*) and recruitment (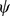), the rate of transition from a non-breeder to a breeding individual. Survival probabilities were assumed to depend on age (*a*), encounter probabilities to be either constant (*c*) or to vary with time (*t*), and recruitment probabilities to depend on age (years after release), time, or be constant across time. *K* is the number of estimated parameters in each model, QAICc is Akaike's information criterion (AIC) adjusted for overdispersion and sample size, ΔQAICc is the difference in QAICc between each model and the best model and *w* denotes the Akaike weights (relative support given to each model).

## Discussion

The use of hand- or captive-reared chicks to reinforce or restore threatened bird populations is now relatively widespread [38]. The approach has been used successfully in combination with translocation in the conservation of at least 11 seabird species worldwide [39]–[42]. However, efforts to restore or reinforce penguin populations appear to be scarce [42], even though the Spheniscidae may represent good candidates species. All members of the family exhibit apparent post-fledging independence, they generally have low levels of parental attendance following the guard stage, and they can be easily hand-fed [43]. Although prolonged hand-feeding of nestlings can reduce fledging success in some seabirds [44], this does not occur with African penguins and, because hand-reared chicks are as fit as naturally-reared chicks [17], [27], the species has been considered a promising candidate for reinforcement and conservation translocation [17].

Our results confirm that the success of hand-rearing African penguin chicks after oiling incidents extends to chicks abandoned by moulting parents. Survival in the first year after release (0.32±0.08) was within the range of estimates for chicks hand-reared after oil spills (0.20–0.42, [17], [45]) and apparent adult survival (0.76±0.10) was also similar to estimates for chicks hand-reared after the 1994 (0.79, [45]) and 2000 oil spills [17]. In addition, juvenile survival compared well to a previous estimate from naturally-reared birds at Robben and Dassen Island from 1987 to 1994 (0.35, [45]) and was towards the upper end of estimates for both juvenile (0.06–0.52) and adult (0.46–0.77) survival at these colonies during our study period [3], [6].

Despite a decreasing breeding population in the Western Cape and poor feeding conditions between 2005 and 2010 [3], an estimated 11% of the hand-reared chicks subsequently recruited into the breeding population. Survival rates measured in this study suggest that around 14% would have survived to breeding age (4 years old [25]). Half of those individuals confirmed as breeding returned to their natal colony, suggesting that this action ultimately acted to reinforce the breeding population at the source colonies [17]. However, removing and hand-rearing African penguin chicks is expensive, labour intensive, and has potential implications for the source populations. Collection of penguin chicks can cause disturbance to moulting adults or other breeding seabirds if not carefully managed. In addition rearing of chicks in captivity exposes them to diseases which could potentially be introduced to wild populations and fledglings may be returned to an environment which cannot support them if prey availability is poor.

### Role of prey availability in chick abandonment

Long-lived birds can alter their reproductive performance according to their body condition and the needs of their offspring [46], choosing not to breed or to abandon a breeding attempt in order to safeguard their own survival [5]. In contrast, moult is obligatory in penguins [24]. It must be undertaken annually and, once initiated, cannot be abandoned prematurely [47], [48]. Thus, the acquisition of insufficient reserves prior to moult compromises survival [21] and the need to exploit a predictable food source during summer – not the fledging of chicks – appears to determine the timing of moult in African penguins [24], [49].

In the Western Cape, moult coincides with the availability of high energy prey [24] while the breeding season is synchronised to the availability of fish in the vicinity of the colonies in winter [50]. During good years, African penguins can successfully rear two broods in a season, but chick growth rates show high plasticity in response to variable feeding conditions [8], [11]. The duration of the fledging period varies as a function of both the local foraging conditions and the energy that parents can afford to invest in chick provisioning [8], [48]. Thus, we hypothesise that the date of egg-laying in the nests which produced the abandoned chicks was early enough to produce fledglings in most years but, in 2006 and 2007, the chicks exhibited such slow growth that they were still nestlings at a point when their parents could no longer delay the initiation of moult. Very slow growth rates were observed at Dyer Island in subsequent years [11] and an increase in fledging periods, similar to that observed at Robben Island [8], may well have occurred. Prey availability in the Western Cape was relatively poor in both 2006 and 2007 [3], such that abandonments could have been mediated either by poor food availability close to the colonies during chick-rearing, poor availability of adult fish during the preceding pre-breeding period, or a combination of the two [8].

African penguins exhibit some natal philopatry [26] and half of the birds breeding in this study returned to their natal site. This was despite evidence that juvenile African penguins may emigrate to non-natal colonies if the food environment is heterogeneous [3], [5], and apparently poor conditions for breeding penguins at Dyer Island in recent years [12], [51] However, if the poor prey availability persists, their subsequent survival and reproductive success would be compromised relative to birds at colonies where conditions are more favourable [3], [6], [8]. As the situation for African penguin has continued to deteriorate on the West Coast [3], [6], plans have been developed to use conservation translocations to establish new breeding colonies in areas of higher prey availability along the South African coast [52].

Our results suggest abandoned chicks as an obvious source of birds for such an endeavour, but the split in recruitment to natal and non-natal sites in birds from Dyer Island suggests that natal imprinting in African penguins occurs before fledging. Nevertheless, translocated individuals will undertake some prospecting behaviour to evaluate the quality of their new habitat, relative to that available to the rest of the meta-population [53]. As such, return rates to translocation sites might well be higher if those sites can be placed in areas perceived to be of high habitat quality or prey availability [53]. The current approach of rearing chicks at SANCCOB to release back at existing colonies (natal and non-natal) provides an opportunity to better understand the dispersal and recruitment process of African penguins [17]. In future, consideration should to be given to whether more could be gained by employing alternative strategies to maximise the conservation benefit of translocations. Rearing birds *in situ* at future release sites has yielded high success rates in chick translocation projects with Procellariiformes [42]. However, this approach comes with additional logistical and financial costs, as well as different risks of disease introduction and environmental impacts. In addition, it may not be necessary for all seabird species, as little penguins *Eudyptula minor* have been successfully translocated by simply keeping them overnight at a release site in artificial nest boxes (N. Carlile, pers. comm.).

### Veterinary concerns

The hand-reared chicks were susceptible to various conditions, in part due to being in captivity (pododermatitis), at high-density (airsacculitis and pneumonia, avian pox) and being exposed to vectors transmitting disease (avian pox, avian malaria).

Pododermatitis can be avoided through the use of varied substrate levels and textures and by having birds regularly walk through disinfectant baths; however, these techniques are generally incompatible with the logistics of large-scale captive rearing. The condition generally improved once the birds were swimming and spending less time standing and does not pose a risk to wild populations.

The severity of avian pox varies between species [36], [54] and the symptoms seen in African penguins are mild to moderate, although mortality of Magellanic penguin *S. magellanicus* chicks has occurred [55]. Prevention of the disease involves control of the vector, isolating heavily infected birds and thorough disinfection of pens, equipment and clothing [36]. It is unlikely to pose a risk to wild populations after release as the lesions resolved over time, although outbreaks can occur in the wild dependent on vector occurrence.

Infections of avian malaria are an ongoing concern at SANCCOB [16]. Avian malaria is present at a low prevalence in wild African penguins [56], [57] although the possibility exists of spreading a pathogenic species from rehabilitated birds into the wild population [29]. This risk is reinforced by the identification of potential vectors on some of the offshore islands (SANCCOB unpubl. data). The incidence of avian malaria at the facility has been dramatically reduced since the erection of new shade cloth netting in 2008 (SANCCOB unpubl. data).

Fungal airsacculitis and pneumonia (most likely to be caused by Aspergillus sp.) occasionally causes deaths in wild African penguin chicks (SANCCOB unpubl. data) and is likely to be more widespread than reported. This is not a condition in released birds that poses a threat to the wild population due to the ubiquitous nature of the organism where infections generally occur secondarily to an immunosuppressive event [54].

While it is possible that releasing large numbers of hand-reared birds into the wild introduced disease into the population [17], [29], [58]–[60] this seems unlikely as surveillance of the colonies is near-continuous and there were no mass mortalities of African penguins during the study period. Sub-clinical diseases remain a possibility [58]–[60], although the comparable subsequent breeding success of hand-reared and naturally-reared African penguins [17] makes this unlikely too. All birds undergo basic disease screening and veterinary evaluation before release in order to reduce any disease introduction risk. A programme of ongoing disease surveillance throughout the breeding range is also recommended to minimise this risk.

Finally, one missing element in the strategy for chick removal in this study was quantitative criteria to decide whether individual chicks were in sufficiently poor condition to conclude that they had been abandoned. The development and use of a body condition index for African penguin chicks [13] provides the opportunity to relate chick condition at admission to survival and to generate adaptive decision rules about the need for chick removal, and its timing in future.

### Conclusions

Hand-rearing of African penguin chicks is a valuable conservation tool in light of the declining population. Continued monitoring of body condition in penguin chicks should be a priority in the management of colonies to ensure the timely collection of abandoned chicks. Further research on the relationship between these abandonment events and variations in prey availability at different temporal and spatial scales is warranted and a programme of disease surveillance is recommended to help limit any possibility of disease outbreak. Finally, additional research on how the dispersal of fledging African penguins relates to prey availability could pave the way for successful conservation translocations to establish new colonies in favourable breeding localities for this ‘Endangered’ species.

## Supporting Information

Appendix S1 **Additional information on the chick-rearing methods and results: (a) the system used to classify chicks by stage of development, (b) the system used to classify chicks by Habitus, (c) the basic treatment protocols used during hand-rearing.**
(PDF)

Appendix S2 **The state-transition and observation matrices for the multistate models.**
(PDF)

Appendix S3 **Data on the African penguin chicks admitted to SANCCOB in 2006 and 2007.**
(XLSX)

Table S1 **Numbers of African penguin chicks admitted to SANCCOB from 2001 to 2005.**
(PDF)

Table S2 **Numbers of African penguin chicks removed in 2006 and 2007, compared to the number of breeding pairs at Dyer Island, Robben Island and Stony Point.**
(PDF)

Table S3 **Numbers of abandoned African penguin chicks released according to area of origin and area of release, with number of banded individuals.**
(PDF)

Table S4 **Numbers of African penguin chicks which were positive for avian malaria.**
(PDF)

Table S5 **Additional information on the 12 hand-reared chicks observed breeding by December 2012.**
(PDF)
