# Supplementary material for: Hand-Rearing, Release and Survival of African Penguin Chicks Abandoned Before Independence by Moulting Parents
Source: PLoS One. 2014 Oct 22;9(10):e110794. doi: 10.1371/journal.pone.0110794 (PMC4206437; doi:10.1371/journal.pone.0110794)
Supplement: Appendix S1 — Additional information on the chick-rearing methods and results: (a) the system used to classify chicks by stage of development, (b) the system used to classify chicks by Habitus, (c) the basic treatment protocols used during hand-rearing. (PDF) [file pone.0110794.s001.pdf]

Appendix S1: Additional information on the chick-rearing methods and results.

(a) Stage of development classification

Chicks were sorted by their approximate size by classifying them into five stages of development, following Barham et al. [1,2] and based on the ontogeny described by Seddon and van Heezik [3]:

P0 – newly hatched chicks, naked, eyes generally closed (< six days old [3]);
P1 – small downy chicks, eyes open, unable to sit but can hold their head steady, in the field generally still covered by the adult (about six to 15 days old [3]);
P2 – medium to large downy chicks, from about 50 to 75% of adult size, secondary down on the belly becomes white, able to sit, in the field may sit beside the adult or lie partially covered beneath the adult (about 16 to 35 days old [3]);
P3 – large downy chicks, at least 75% of adult size, first evidence of loss of down (usually on the face and flippers) but less than 50% of their body covered with final fledging plumage (about 36 to 45 days old [3][1]);
P4 – chicks with more than half of their body covered with final fledging plumage (> 45 days old [3]).

(b) Habitus classification

Habitus is an expert evaluation of the condition of the bird on a scale of 1–4 [4]:

1 – bird is weak, not able to stand and not aware of its surroundings;
2 – bird is weak, able to stand, but still not aggressive and lies down a lot;
3 – bird is stronger, stands, aggressive when handled and;
4 – bird is strong and very aggressive.

(c) Basic treatment protocols

Basic treatment protocols used at SANCCOB during 2006 and 2007 are listed below but treatments were not necessarily limited to these options, nor were they always instituted but each case managed on an individual basis. Treatment protocols are only listed for those conditions discussed in the text. All treatments were given orally.

Avian malaria – Chloroquine 10 mg/kg 0hr, 5 mg/kg 6, 18 and 24 hr, and
Primiquine 1 mg/kg once daily (o.i.d.) for 10 days or as long as needed.

Systemic antibiotics used for airsacculitis and pneumonia, avian pox and bumblefoot lesions –
Enrofloxacin 20 mg/kg twice daily (b.i.d.) for 10 days or as long as needed,
or
Clavulanate potentiated Amoxycillin 100 mg/kg three times a day (t.i.d.) for 10 days or as long as needed, or

Amoxycillin 200 mg/kg t.i.d. for 10 days or as long as needed, or

Trimethoprim Sulphamethoxazole 40 mg/kg b.i.d. for 10 days or as long as needed.

Anti-inflammatory used for avian pox and bumblefoot lesions –

Meloxicam 0.2 mg/kg o.i.d. for 10 days or as long as needed.

Anti-fungal – Itraconazole10 mg/kg b.i.d. for 2–3 weeks.

References

1. Barham PJ, Underhill LG, Crawford RJM, Leshoro TM (2007) Differences in breeding success between African penguins (Spheniscus demersus) that were and were not oiled in the MV Treasure oil-spill in 2000. Emu 107: 7–13. doi:10.1071/MU06028.
2. Barham PJ, Underhill LG, Crawford RJM, Altwegg R, Leshoro MT, et al. (2008) The efficacy of hand-rearing penguin chicks: evidence from African Penguins (Spheniscus demersus) orphaned in the Treasure oil spill in 2000. Bird Conserv Int 18: 144–152. doi:10.1017/S0959270908000142.
3. Seddon PJ, van Heezik YM (1993) Behaviour of the jackass penguin chick. Ostrich 64: 8–12. doi:10.1080/00306525.1993.9634188.
4. Parsons NJ, Underhill LG (2005) Oiled and injured African penguins Spheniscus demersus and other seabirds admitted for rehabilitation in the Western Cape, South Africa, 2001 and 2002. Afr J Mar Sci 27: 289–296. doi:10.2989/18142320509504087.
