# Supplementary material for: Hand-Rearing, Release and Survival of African Penguin Chicks Abandoned Before Independence by Moulting Parents
Source: PLoS One. 2014 Oct 22;9(10):e110794. doi: 10.1371/journal.pone.0110794 (PMC4206437; doi:10.1371/journal.pone.0110794)
Supplement: Appendix S2 — The state-transition and observation matrices for the multistate models. (PDF) [file pone.0110794.s002.pdf]

Appendix S2: The state-transition and observation matrices for the multistate models describing survival and recruitment in the hand-reared chicks.

The state-transition matrix from the ‘true’ states at *t* (in rows) to the ‘true’ state at *t+1* (in columns) is described by the survival (ϕ) and recruitment (ψ) parameters:

	**‘Alive NB’**	**‘Alive B’**	**‘Dead’**
**‘Alive NB’**	$\phi^{NB} 1-\psi^{NB,B}$	$\phi^{NB}\psi^{NB,B}$	$1-\phi^{NB}$
**‘Alive B’**	$\phi^{B}\psi^{B,NB}$	$\phi^{B} 1-\psi^{B,NB}$	$1-\phi^{B}$
**‘Dead’**	0	0	1

Where ϕ^{NB} and ϕ^{B} are the survival probabilities for non-breeding birds (NB) and breeding birds (B) respectively. $\psi^{NB,B}$ denotes the probability of transitioning from being a non-breeder to being a breeder (the recruitment probability) and $\psi^{B,NB}$ the probability of transitioning back from being a breeder to being a non-breeder. In essence, because birds may choose to breed or not in any given year once they have reached maturity, this transition probability may reflect either breeding absenteeism or birds that bred but where not encountered.

In turn, the observation (or event) matrix maps the ‘true’ states at *t* (in rows) to the observed states at *t* (in columns) as follows:

	**‘Not encountered’**	**‘Encountered as NB’**	**‘Encountered as B’**
**‘Alive NB**	$1-p^{NB}$	p^{NB}	0
**‘Alive B’**	$1-p^{B}$	0	p^{B}
**‘Dead’**	1	0	0

Where p^{NB} and p^{B} are the encounter probabilities of non-breeders (NB) and breeders (B) respectively. For details on the candidate models tested, see the main text.
