# Supplementary material for: Hand-Rearing, Release and Survival of African Penguin Chicks Abandoned Before Independence by Moulting Parents
Source: PLoS One. 2014 Oct 22;9(10):e110794. doi: 10.1371/journal.pone.0110794 (PMC4206437; doi:10.1371/journal.pone.0110794)
Supplement: Table S1 — Numbers of African penguin chicks admitted to SANCCOB from 2001 to 2005. (PDF) [file pone.0110794.s004.pdf]

Table S1. Numbers of African penguin chicks admitted to SANCCOB after being abandoned by moulting parents on Robben Island, Dyer Island and at Stony Point, 2001-2005.

Year	Colony	No. admitted	No. released	Release rate
2001	Robben Island	32	10	31%
	Dyer Island	1	0	0%
2002	Robben Island	24	10	42%
2003	Robben Island	4	2	50%
	Dyer Island	81	48	59%
2004	Robben Island	6	3	50%
	Dyer Island	93	49	53%
2005	Robben Island	3	0	0%
	Dyer Island	57	37	65%
**Total**		301	159	53%
