# Supplementary material for: Hand-Rearing, Release and Survival of African Penguin Chicks Abandoned Before Independence by Moulting Parents
Source: PLoS One. 2014 Oct 22;9(10):e110794. doi: 10.1371/journal.pone.0110794 (PMC4206437; doi:10.1371/journal.pone.0110794)
Supplement: Table S2 — Numbers of African penguin chicks removed in 2006 and 2007, compared to the number of breeding pairs at Dyer Island, Robben Island and Stony Point. (PDF) [file pone.0110794.s005.pdf]

Table S2. The number of African penguin chicks removed from September to December 2006 and 2007, compared to the number of breeding pairs at Dyer Island, Robben Island and Stony Point.

Colony	Year	No. chicks removed	No. breeding pairs	chick/breeding pair
Dyer Island	2006	694	2057	0.34
	2007	427	1513	0.28
Robben Island	2006	113	3697	0.03
	2007	7	5935	0.001
Stony Point	2006	34	265	0.13
	2007	47	260	0.18

The number of breeding pairs is based on annual count data presented in Crawford et al. 2011, reference [3] in the main text.
