# Supplementary material for: Hand-Rearing, Release and Survival of African Penguin Chicks Abandoned Before Independence by Moulting Parents
Source: PLoS One. 2014 Oct 22;9(10):e110794. doi: 10.1371/journal.pone.0110794 (PMC4206437; doi:10.1371/journal.pone.0110794)
Supplement: Table S3 — Numbers of abandoned African penguin chicks released according to area of origin and area of release, with number of banded individuals. (PDF) [file pone.0110794.s006.pdf]

Table S3. Numbers of abandoned African penguin chicks admitted to SANCCOB between September and December, 2006 and 2007, subsequently released according to area of origin and area of release, with number of banded individuals.

Year	Colony of Origin	Colony of Release	No. birds	No. banded birds
2006	Stony Point	Robben Island	29	29
	Dyer Island	Dyer Island	322	207
		Robben Island	325	215
	Robben Island	Robben Island	90	60
2007	Stony Point	Dyer Island	12	4
		Robben Island	12	2
	Dyer Island	Dyer Island	201	158
		Robben Island	123	24
	Robben Island	Dyer Island	3	2
**Total**			1117	701
