# Supplementary material for: Hand-Rearing, Release and Survival of African Penguin Chicks Abandoned Before Independence by Moulting Parents
Source: PLoS One. 2014 Oct 22;9(10):e110794. doi: 10.1371/journal.pone.0110794 (PMC4206437; doi:10.1371/journal.pone.0110794)
Supplement: Table S4 — Numbers of African penguin chicks which were positive for avian malaria. (PDF) [file pone.0110794.s007.pdf]

Table S4. Numbers of African penguin chicks admitted for hand-rearing at SANCCOB, released or that died between September and December 2006 and 2007 which were positive for avian malaria and percentages of the total in each case.

	**2006**			**2007**		
	Admissions	Releases	Deaths	Admissions	Releases	Deaths
No. birds	114	81	33	112	42	70
% of category	14%	11%	43%	23%	12%	54%
Mean ± SD duration (d)	–	58 ± 23	53 ± 26	–	81 ± 27	58 ± 26

The mean ± standard deviation (SD) duration in days (d) from admission to release or death is also shown.
