# Supplementary material for: Hand-Rearing, Release and Survival of African Penguin Chicks Abandoned Before Independence by Moulting Parents
Source: PLoS One. 2014 Oct 22;9(10):e110794. doi: 10.1371/journal.pone.0110794 (PMC4206437; doi:10.1371/journal.pone.0110794)
Supplement: Table S5 — Additional information on the 12 hand-reared chicks observed breeding by December 2012. (PDF) [file pone.0110794.s008.pdf]

Table S5. Additional information on the 12 hand-reared chicks that had recruited into the breeding population by December 2012.

**SAFRING Number**	**Date of capture**	**Origin**	**Release date**	**Release method**	**Release site**	**First resighting date**	**Breeding at**	**No. times seen breeding**	**Age at first breeding (years)**
A12154	17 Oct. 2006	DYI	19 Dec. 2006	B	ROI	28 May 2009	ROI	1	2.4
A12430	17 Oct. 2006	DYI	15 Nov. 2006	C	ROI	04 May 2010	ROI	4	3.5
A12431	17 Oct. 2006	DYI	09 Nov. 2006	C	ROI	10 Apr. 2010	ROI	1	3.4
A12448	17 Oct. 2006	DYI	09 Nov. 2006	C	ROI	20 Jul. 2009	DYI	2	2.7
A12766	17 Oct. 2006	DYI	21 Nov. 2006	C	DYI	01 Jun. 2008	DYI	1	1.5
A12787	21 Oct. 2006	DYI	21 Nov. 2006	C	DYI	03 Apr. 2010	DYI	1	3.4
A12803	21 Oct. 2006	DYI	05 Dec. 2006	B	ROI	08 Apr. 2011	DAI	5	4.3
A12820	21 Oct. 2006	DYI	28 Nov. 2006	C	DYI	27 Aug. 2009	DYI	1	2.7
A12858	21 Oct. 2006	DYI	28 Nov. 2006	C	DYI	29 Aug. 2011	STP	1	4.8
A12953	21 Oct. 2006	DYI	28 Nov. 2006	C	DYI	26 Apr. 2010	DYI	1	3.4
A12988	21 Oct. 2006	DYI	28 Nov. 2006	C	DYI	17 Mar. 2011	STP	3	4.3
A12997	17 Oct. 2006	DYI	28 Nov. 2006	C	DYI	29 May 2011	DYI	1	4.5

Abbreviations: SAFRING = South African Bird Ringing Unit; DYI = Dyer Island; ROI = Robben Island; STP = Stony Point; DAI = Dassen Island; B = released from a boat just offshore of Robben Island; C = released on land at the colony listed under Release site.
